# Increasing Incidence and Age at Diagnosis among Children with Type 1 Diabetes Mellitus over a 20-Year Period in Auckland (New Zealand)

**DOI:** 10.1371/journal.pone.0032640

**Published:** 2012-02-28

**Authors:** José G. B. Derraik, Peter W. Reed, Craig Jefferies, Samuel W. Cutfield, Paul L. Hofman, Wayne S. Cutfield

**Affiliations:** 1 Liggins Institute, University of Auckland, Auckland, New Zealand; 2 Starship Children's Health, Auckland District Health Board, Auckland, New Zealand; 3 National Research Centre for Growth and Development, University of Auckland, Auckland, New Zealand; Johns Hopkins Bloomberg School of Public Health, United States of America

## Abstract

**Background:**

We aimed to evaluate the incidence of type 1 diabetes mellitus in children <15 years of age (yr) in the Auckland region (New Zealand) over 20 years (1990–2009).

**Methods:**

We performed a retrospective review of all patients <15 yr diagnosed with type 1 diabetes, from an unselected complete regional cohort.

**Results:**

There were 884 new cases of type 1 diabetes, and age at diagnosis rose from 7.6 yr in 1990/1 to 8.9 yr in 2008/9 (r^2^ = 0.31, p = 0.009). There was a progressive increase in type 1 diabetes incidence among children <15 yr (p<0.0001), reaching 22.5 per 100,000 in 2009. However, the rise in incidence did not occur evenly among age groups, being 2.5-fold higher in older children (10–14 yr) than in the youngest group (0–4 yr). The incidence of new cases of type 1 diabetes was highest in New Zealand Europeans throughout the study period in all age groups (p<0.0001), but the rate of increase was similar in New Zealand Europeans and Non-Europeans. Type 1 diabetes incidence and average annual increase were similar in both sexes. There was no change in BMI SDS shortly after diagnosis, and no association between BMI SDS and age at diagnosis.

**Conclusions:**

There has been a steady increase in type 1 diabetes incidence among children <15 yr in Auckland over 20 years. Contrary to other studies, age at diagnosis has increased and the greatest rise in incidence occurred in children 10–14 yr. There was little change in BMI SDS in this population, providing no support for the ‘accelerator hypothesis’.

## Introduction

The incidence of type 1 diabetes mellitus has been increasing worldwide [1], [2], [3], and it appears to have been particularly pronounced among children <5 years of age (yr) [3], [4], [5]. This increase has been suggested to be associated with the ‘accelerator hypothesis’ [6]. Although this hypothesis is not universally accepted [7], it predicts that higher BMI is associated with younger age at type 1 diabetes diagnosis [8], which has been demonstrated in some studies [9], [10], [11].

New Zealand has a population of approximately 4.4 million people, the majority being of European descent. Auckland, the largest city in New Zealand, is the most ethnically diverse, with approximately 11% of people identifying themselves as indigenous Maori, 14% as Pacific, and 19% as Asian [12]. By international standards, the incidence of type 1 diabetes in young New Zealanders was assessed as moderate at 17.9 per 100,000 [13]. However, this figure was obtained from a 2-year snapshot, and did not provide information on possible time trends on type 1 diabetes incidence. In addition, previous studies on type 1 diabetes incidence in New Zealand are out of date or refer to a specific geographical region [14], [15], [16].

This study aimed to determine: i) whether the incidence of type 1 diabetes in children under 15 yr in the Auckland region has changed over the last 20 years; ii) how incidence differs between gender, age and ethnicity groups; and iii) to assess whether any changes are consistent with the ‘accelerator hypothesis’.

## Methods

Ethics approval was provided by the Auckland District Health Board Research Review Committee (ADHB-RRC). Written or verbal informed consent was not required for ethics approval, as this study involved an audit of routine clinical practice.

The Endocrinology Service at Starship Children's Health provides specialist care for all children diagnosed with type 1 diabetes in the Auckland region (New Zealand). Its Paediatric Diabetes Service provides centralised medical care for all diabetic children up to 15 yr who reside in the Auckland region, drawing from the regional population of approximately 1.5 million [12]. All children or adolescents diagnosed with type 1 diabetes who attended the Paediatric Service between 1 January 1990 and 31 December 2009 were eligible for this study. Subjects were captured from a comprehensive database (Starbase) that gathers data on all children with type 1 diabetes in the Auckland region. This information was cross-referenced with hospital admission data and subsequent clinical follow up, leading to a case ascertainment >95% for children with type 1 diabetes [13].

Only children aged <15 yr were included. Type 1 diabetes was diagnosed based on clinical features. All patients had elevated blood glucose at presentation: either a random measurement of ≥11.1 mmol/l and presence of classical symptoms, or fasting blood glucose ≥7.1 mmol/l. In addition, all patients met at least one of the following criteria: a) diabetic ketoacidosis; b) presence of at least two type 1 diabetes antibodies (to glutamic acid decarboxylase, islet antigen 2, islet cell, or insulin autoantibodies); or c) ongoing requirement for insulin therapy. Clinical and demographic data were prospectively recorded on all patients at each outpatient visit.

From 1994 onwards, anthropometric data were recorded at each clinic visit, and for the purposes of this study we used data from the first post-diagnosis clinic that usually occurred 3–4 months afterwards. Standard deviation scores (SDS) were calculated based on the British 1990 Growth Reference Data [17] to obtain height SDS, weight SDS, and body mass index (BMI) SDS.

Ethnicity was recorded by self-report using a prioritised system, such that if multiple ethnicities were selected, the patient was assigned to a single category, following a hierarchical system of classification [18]. Patients were assigned to European, Maori, Pacific Islander, or Other (Asian/Middle Eastern/Latin American/African) groups, which match national census classifications.

To assess whether changes in incidence were more marked in certain age groups (as observed overseas [3], [4]), patients were also categorised into three bands according to age at diagnosis: 0–4 yr (children less than 5 yr), 5–9 yr (equal or greater than 5 yr but less than 10 yr), and 10–14 yr (equal or greater than 10 yr but less than 15 yr). These age bands also match national census classifications. The incidence of type 1 diabetes was assessed as the number of new diagnoses per 100,000 age-matched inhabitants on a given year, based on the 5-yearly national census data from Statistics New Zealand [12] and interpolated estimates of the population for the intervening years. Incidence was modelled using the Poisson distribution. Point estimates were calculated with exact Poisson confidence limits, and change in incidence over time were analysed using Poisson regression. Changes in patient numbers, age at diagnosis, and anthropometric data over time were assessed by linear regression. Poisson modelling was undertaken using StatsDirect v2.7.8 (StatsDirect Ltd, UK); other analyses were undertaken using JMP v. 5.1 (SAS Inc, USA).

## Results

A total of 884 new patients aged <15 yr were diagnosed with type 1 diabetes over the 20-year period covered by this study. There was an increase in the mean age at diagnosis from 7.6 yr in 1990/1 to 8.9 yr in 2008/9 (0.07/yr, r^2^ = 0.31, p = 0.009). This was observed in both males (0.07/yr, r^2^ = 0.22, p = 0.04) and females (0.06/yr, r^2^ = 0.13, p = 0.12).

There was a steady increase in the annual number of newly diagnosed cases of type 1 diabetes in children <15 yr (r^2^ = 0.80; p<0.0001) of 2.0 additional cases per year, from 23 in 1990/1 to 60 cases per year in 2008/9. There was no appreciable difference in the rate of increase between males and females (p = 0.08), but the rise in number of new type 1 diabetes cases did not occur evenly among age groups (p = 0.0001). The yearly increase among older children (10–14 yr) was 3-fold greater than in the youngest (0–4 yr) group (0–4 yr = +0.4/yr; 5–9 yr = +0.8/yr; 10–14 yr = +1.2/yr). Over the 20-year period, new cases were moderately more frequent in winter and less frequent in spring (29.4% and 22.0%, respectively; test of equal proportions across all four seasons: p = 0.02).

**Figure 1:**
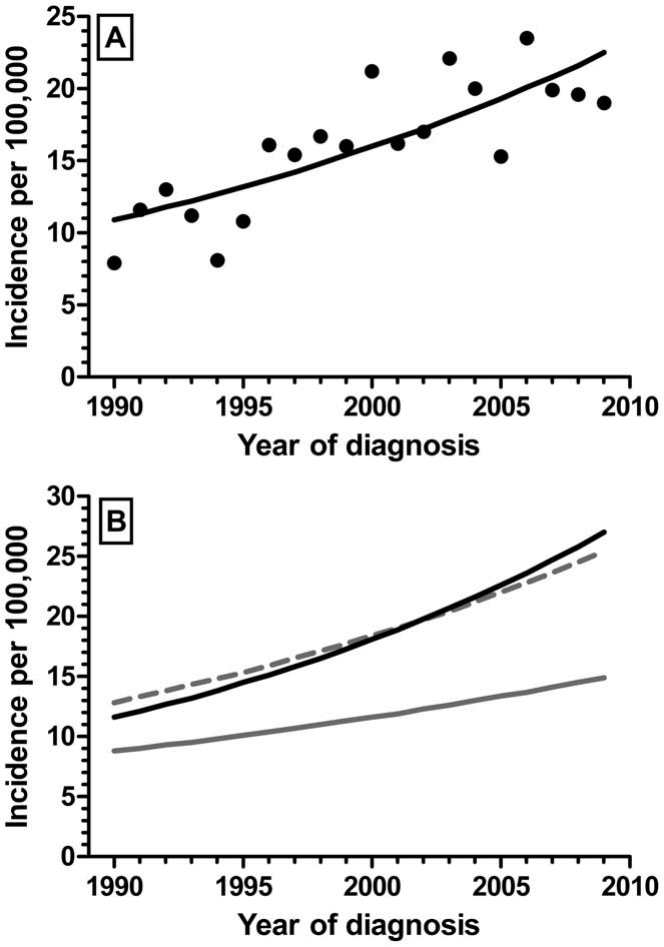
Incidence of new cases of type 1 diabetes mellitus per 100,000 age-matched inhabitants diagnosed in Auckland (New Zealand) during 1990–2009. A) For all children <15 yr; B) for children 0–4 yr (solid gray line), 5–9 yr (dashed gray line), and 10–14 yr (solid black line).

The annual incidence of type 1 diabetes in children <15 yr in the Auckland population in 1990–2009 was 16.4/100,000 (95% CI 15.3–17.5). Considering the underlying 36% population growth over the 1990–2009 period, there was still a progressive increase in the incidence of new cases (p<0.0001; Figure 1A). By Poisson regression the type 1 diabetes incidence in children <15 yr in 2009 was 22.5 per 100,000 (95% CI 17.5–28.4), in comparison to 10.9 per 100,000 in 1990 (95% CI 7.0–16.1) (Figure 1A). Overall incidence among males and females across the 20-year period was similar (p = 0.49). The increase in incidence was greatest among children 10–14 yr (average increase of +0.81/year; p<0.0001) and lowest among children 0–4 yr (+0.32/year; p = 0.02); incidences by 2009 were 27.0 (95% CI 18.1–38.8) for children 10–14 yr, 25.4 (95% CI 16.5–37.3; +0.66/year; p = 0.0002) for children 5–9 yr, and 14.9 per 100,000 (95% CI 8.4–24.5) for those aged 0–4 yr (Figure 1B).

**Figure 2:**
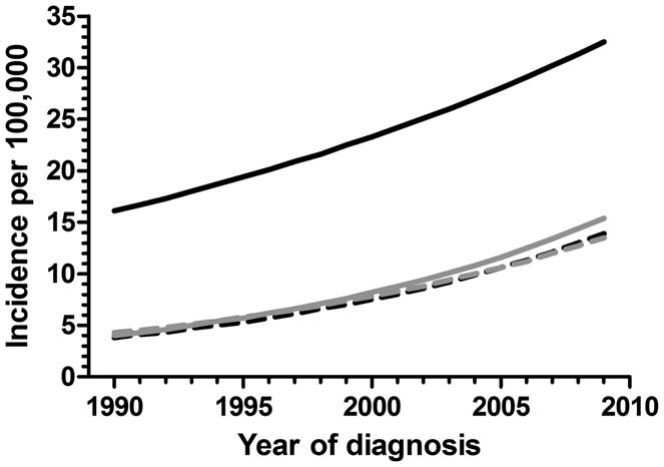
Incidence of new cases of type 1 diabetes mellitus per 100,000 age-matched inhabitants diagnosed in Auckland (New Zealand) among different ethnic groups during 1990–2009: New Zealand Europeans (solid black line), Maori (dashed black line), Pacific Islanders (solid gray line), Others (dashed gray line).

The incidence of type 1 diabetes was higher in New Zealand Europeans than other ethnic groups throughout the study period (Figure 2, p<0.0001). There was little difference in incidence among non-European ethnic groups. The annual incidences (per 100,000) by 2009 were: Europeans 32.5 (95% CI 23.8–43.3), Non-Europeans 14.4 (95% CI 9.2–21.4), Maori 13.9 (95% CI 5.2–29.7), Pacific Islanders 15.4 (95% CI 7.3–28.5), and Other 13.5 (95% CI 5.8–26.8). The rate of increase in incidence over the study period was very similar across all ethnicities, as illustrated by the slopes in Figure 2. However, while the average increase in incidence was higher for Europeans than Non-Europeans in children of all age groups (Table 1), the increase was proportionally lower in Europeans (2-fold) than Non-Europeans (3-fold) due to a lower baseline incidence in the latter group (Figure 2). Nonetheless, in both ethnic groups type 1 diabetes incidence in children 10–14 yr increased at a higher rate than in the youngest 0–4 yr group, with a >2-fold difference observed among both Europeans and Non-Europeans (Table 1). Age at diagnosis across the study period was similar in both ethnic groups (p = 0.47).

**Table 1:** Changes in the age-specific incidence of type 1 diabetes mellitus per 100,000 in children <15 years of age (yr) in the Auckland region in 1990–2009.

Age band	Europeans	Non-Europeans
0–4 years	+0.5/yr (r^2^ = 0.21; p = 0.04)	+0.3/yr (r^2^ = 0.24; p = 0.04)
5–9 years	+0.9/yr (r^2^ = 0.24; p = 0.002)	+0.7/yr (r^2^ = 0.60; p = 0.0006)
10–14 years	+1.2/yr (r^2^ = 0.31; p = 0.0001)	+0.7/yr (r^2^ = 0.54; p = 0.001)
<15 years	+0.9/yr (r^2^ = 0.45; p<0.0001)	+0.5/yr (r^2^ = 0.77; p<0.0001)

Height and weight were recorded for 660 patients at their required first post-diagnostic clinic (on average 15 weeks from diagnosis) from 1994 onwards. Annual mean BMI SDS of newly diagnosed type 1 diabetes did not alter (average non-significant change smaller than ±0.02 SDS/year) over the period for the entire population, or for any gender, age, or ethnicity sub-group. There was no association between BMI SDS and age at diagnosis.

## Discussion

This study shows that the incidence of type 1 diabetes in the Auckland region has increased steadily over the last two decades. However, unlike other studies [3], [4], [5], the rate of increase in incidence has been particularly marked in older children (10–14 yr), which was approximately 2.5-fold greater than that in children 0–4 yr. Interestingly, the incidence of type 1 diabetes in children 0–4 and 10–14 in Auckland are very similar to those reported in Australia, our closest geographical and ethnic neighbours [19], both of which had very high case ascertainment levels (close to 100%).

The reasons underpinning the considerable increase in incidence over the study period are unclear. This may reflect an actual change in the type 1 diabetes incidence in patients <15 yr. Alternatively, it may reflect an earlier age of onset without change in incidence over all ages, so that greater numbers of people are being diagnosed with type 1 diabetes in adolescence rather than in young adulthood. This would be consistent with the ‘accelerator hypothesis’, which suggests that an increasing rate of obesity is a primary driver for an earlier age of diabetes onset [6]. Studies have shown an association between higher BMI and younger age at diagnosis [9], [10], [11], indicating greater adiposity in childhood may hasten the onset of diabetes mellitus. The ‘accelerator hypothesis’ predicts an early onset rather than increased risk [11], and a Swedish study examining type 1 diabetes incidence on a nation-wide cohort 0–34 yr showed a shift in age of onset towards younger ages, rather than an increase in incidence *per se* across the whole population [20]. Although we cannot rule out a similar phenomenon in Auckland, we did not observe an increase in BMI SDS among children recently diagnosed with type 1 diabetes, or an association between BMI SDS and age at diagnosis. In fact, we observed an actual increase in age at diagnosis which is inconsistent with the ‘accelerator hypothesis’. Thus, our data suggest a true increase in the incidence of type 1 diabetes in the Auckland region, and not changes driven by increasing adiposity.

New Zealand Europeans had a significantly higher incidence rate than Non-Europeans, which is consistent with other studies [21], [22]. There was a marked decrease in the proportion of Europeans in Auckland over the study period, so that the increase in type 1 diabetes incidence was not due to a shift in ethnic distribution. Furthermore, the incidence has been increasing in both Europeans and non-Europeans. A number of studies have shown that immigrant groups display higher rates of type 1 diabetes than in their countries of origin, particularly those that move into societies with a westernised lifestyle [23], [24]. For example, although type 1 diabetes in Polynesia is extremely rare, an abrupt increase in incidence occurs in Pacific Island peoples who migrate to New Zealand [25]. Our study provides evidence that the factors leading to an increase in incidence are operating across all ethnicities. Indeed, the incidence of type 1 diabetes has been remarkably similar over time for the indigenous Maori and the largely newly immigrant Pacific Island and Other ethnic groups.

We observed no significant differences between sexes for any of the parameters investigated. Other studies have found similar results, although at least two investigations provided evidence that females are diagnosed with diabetes at a younger age than males [9], [20].

In conclusion, there has been a steady increase in type 1 diabetes incidence in children <15 yr in Auckland over a 20-year span. However, in contrast to observations elsewhere, age at diagnosis in Auckland has increased over the study period. Our data do not support the ‘accelerator hypothesis’, and factors other than simply increasing adiposity are likely to be at play.
